# Anti‐Proliferative Effects of Resveratrol on Gingival Fibroblasts Derived From Amlodipine‐Induced Gingival Overgrowth

**DOI:** 10.1111/jre.70112

**Published:** 2026-04-11

**Authors:** Bilkan Kara, Nuriye Işıl Saygun, Cansel Köse Özkan, Melis Özgül Slezovic, Muhittin Abdulkadir Serdar, Mualla Pınar Elçi, Alpdoğan Kantarci

**Affiliations:** ^1^ Department of Periodontology, Faculty of Dentistry University of Health Sciences Ankara Turkiye; ^2^ Department of Pharmaceutical Technology, Faculty of Pharmacy University of Health Sciences Ankara Turkiye; ^3^ Department of Periodontology, Faculty of Dentistry Lokman Hekim University Ankara Turkiye; ^4^ Department of Medical Biochemistry Acıbadem Mehmet Ali Aydınlar University Ankara Turkiye; ^5^ Faculty of Medicine, Institute of Stem Cell Lab University of Health Sciences Ankara Turkiye; ^6^ Department of Developmental and Surgical Science, School of Dental Medicine University of Minnesota Minneapolis Minnesota USA

**Keywords:** amlodipine, COL‐I, gingival overgrowth, growth factors, resveratrol, SOD, TGM‐2

## Abstract

**Aim:**

This study aimed to investigate the anti‐proliferative effects of resveratrol on gingival fibroblasts derived from amlodipine‐induced gingival overgrowths and its impact on the levels of transforming growth factor‐*β*1 (TGF‐*β*1), connective tissue growth factor (CTGF), collagen type I (COL‐1), interleukin‐6 (IL‐6), transglutaminase‐2 (TGM‐2), and superoxide dismutase (SOD), which play critical roles in collagen and extracellular matrix metabolism in gingival enlargement.

**Methods:**

Primary gingival fibroblasts were isolated from patients with amlodipine‐induced gingival overgrowth (GO group) and from healthy gingival tissues (H group). Each group was divided into untreated and resveratrol‐treated subgroups. Cell proliferation and viability at 24 and 48 h were assessed by MTT assay. The levels of TGF‐*β*1, CTGF, COL‐1, IL‐6, TGM‐2, and SOD were quantified using ELISA.

**Results:**

MTT values were significantly higher in the GO group than in the H group at both 24 and 48 h (*p* = 0.01). Resveratrol treatment significantly decreased proliferation in the GO group at both time points (*p* < 0.001). In the GO group, resveratrol reduced TGF‐*β*1 (*p* = 0.0090), CTGF (*p* = 0.0090), TGM‐2 (*p* = 0.0088), COL‐1 (*p* = 0.0139), and IL‐6 (*p* = 0.050) levels at 24 h and significantly increased SOD levels (*p* = 0.0143).

**Conclusion:**

Resveratrol suppressed cellular proliferation without exerting cytotoxic effects in amlodipine‐induced GO, while downregulating fibrosis markers (TGF‐*β*1, CTGF, COL‐1, IL‐6, and TGM‐2) and restoring diminished SOD synthesis.

**Trial Registiration:**

This study did not involve a clinical trial and therefore registration was not required

## Clinical Relevance

**Background:**

The management of amlodipine‐associated gingival enlargement may involve changing the patient's medication, non‐surgical periodontal therapy, or surgical periodontal treatment. However, the high recurrence rate of drug‐induced gingival overgrowth complicates treatment in cases where altering the patient's medication is not feasible. Therefore, additional therapeutic approaches for drug‐induced gingival enlargement need to be explored.

**Added Value of This Study:**

In our study, repeated administration of resveratrol reduced excessive cell growth without causing cell toxicity and lowered the levels of molecules responsible for tissue fibrosis, such as TGF‐*β*1, CTGF, COL‐1, IL‐6, and TGM‐2. Moreover, it increased the levels of SOD, an important antioxidant enzyme that protects cells from oxidative stress.

**Clinical Implications:**

These findings suggest that resveratrol may help prevent or reduce gingival overgrowth related to amlodipine by balancing oxidative and fibrotic pathways in gingival tissues. It is considered that the use of resveratrol through controlled‐release systems or appropriate delivery vehicles, administered locally or systemically, may provide the basis for developing a non‐invasive treatment model for gingival overgrowth. Nevertheless, randomized long‐term clinical trials are required before resveratrol can be integrated into clinical practice for the treatment of gingival overgrowth in dentistry.

## Graphical abstract

Resveratrol in repeated doses has been shown to suppress excessive cell proliferation without cytotoxic effects in amlodipine‐induced gingival hyperplasia, while not having a negative effect on mediators involved in tissue homeostasis in healthy cells, and at the same time, it reduces the expression of biomarkers involved in fibrosis processes.

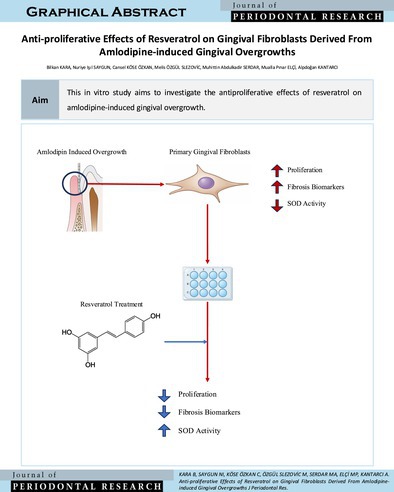

## 1 Introduction

Drug‐induced gingival overgrowth (GO) represents one of the most common forms of gingival hyperplasia [1]. Amlodipine, a calcium channel blocker primarily used in the treatment of hypertension and angina, has been reported to cause GO as an adverse effect [2]. The prevalence of gingival overgrowth in patients receiving amlodipine has been reported to range between 26% and 37% [3, 4]. In calcium channel blocker–induced GO, inflammation and fibroblast proliferation are commonly observed, along with increased collagen levels and disruption of the balance between the synthesis and degradation of the extracellular matrix within the gingival connective tissues [5]. Some of the factors proposed to be responsible for this imbalance include cytokines, inflammatory mediators, and growth factors [6, 7, 8].

It has been reported that the levels of various mediators—including transforming growth factor beta‐1 (TGF‐*β*1), connective tissue growth factor (CTGF), fibroblast growth factor‐2 (FGF‐2), platelet‐derived growth factor‐beta (PDGF‐*β*), interleukin‐6 (IL‐6), interleukin‐1*β* (IL‐1*β*), and reactive oxygen species—are elevated in enlarged gingival tissues [9]. TGF‐*β*1 is considered one of the most important cytokines regulating collagen metabolism. Depending on the target cell type or tissue structure, it can either decrease or increase cellular proliferation, influence differentiation, or play a role in various fibrotic diseases associated with extracellular matrix (ECM) accumulation [10]. CTGF is an important signaling molecule involved in various biological processes such as cell proliferation, angiogenesis, and wound healing, as well as in pathological conditions including tumor development, stimulation of ECM accumulation and remodeling, and the induction of fibrosis [11]. A recent study demonstrated that cells derived from tissues exhibiting GO associated with calcium channel blocker use showed higher levels of CTGF and TGF‐*β* compared with healthy gingival fibroblasts [12]. Another study showed that gingival fibroblasts derived from a patient with juvenile hyaline fibromatosis exhibited higher levels of CTGF and TGF‐*β* compared to controls [13]. Transglutaminase 2 (TGM2) is a multifunctional enzyme that contributes to wound‐healing processes and has been shown to exert fibrotic effects in various organs and tissues, including the liver, kidneys, lungs, and heart [14]. TGM2 also plays an important role in the fixation and activation of the profibrotic cytokine TGF‐*β*. IL‐6 is a cytokine involved in inflammation, immune response, and hematopoiesis, and is known to enhance extracellular matrix production in gingival fibroblasts [6, 15].

In drug‐induced GO, the fibrotic appearance of gingival tissues is determined by imbalances between the synthesis and degradation of extracellular matrix proteins, including type I collagen [16]. Oxidative stress may play a critical role in the onset and progression of fibrotic diseases [17, 18] and there is an association between oxidative stress and gingival overgrowth. Superoxide dismutase (SOD) is a well‐known cellular antioxidant enzyme, reported to function as an aerobic enzyme that reduces toxicity caused by oxidative stress and thereby regulates cell survival. Decreased SOD activity has been observed in systemic fibrotic conditions affecting various organs [19].

Periodontal management of hyperplasias involves reducing inflammation through scaling and root planing, maintaining good oral hygiene, and, if necessary, surgical removal of excess gingival tissue [20]. In drug‐induced cases, surgical intervention is often required; however, many patients' systemic conditions may not be suitable for such procedures. Moreover, in a substantial proportion of patients, modification or discontinuation of the causative medication is not feasible, which may contribute to recurrent gingival overgrowth following surgical treatment. Therefore, there is a need to develop new adjunctive therapeutic strategies for these patients [2, 3]. Resveratrol is a polyphenolic compound characterized by anti‐inflammatory, antimicrobial, anticancer, cardioprotective, vasoprotective, and anti‐fibrotic effects in various organs, as well as antioxidant properties that directly counteract excessive production of reactive oxygen species and restore redox balance [21]. Studies have demonstrated the beneficial effects of resveratrol on periodontal tissues [22, 23]. Recent research reporting the anti‐fibrotic properties of resveratrol in several organs, including the heart, lungs, kidneys, and liver, has highlighted the potential use of this polyphenol in developing a non‐invasive therapeutic model for fibrotic tissues [24, 25, 26]. The anti‐proliferative effect of resveratrol on gingival fibromatosis was first demonstrated on gingival fibroblasts obtained from a patient with juvenile hyaline fibromatosis [13].

Therefore, we tested the hypothesis that resveratrol reverses fibrosis‐associated mechanisms in amlodipine‐induced gingival overgrowth (GO) fibroblasts, thereby providing a potential non‐surgical therapeutic strategy to reduce recurrent gingival enlargement in this common drug‐induced condition. Accordingly, this study aimed to examine how resveratrol affects the levels of key mediators involved in collagen and ECM metabolism, including profibrotic growth factors such as TGF‐*β*, CTGF, and type I collagen (COL‐1), IL‐6, TGM‐2, and SOD, in amlodipine‐induced GO.

## 2 Methods

### 2.1 Cell Culture

Ethical approval was obtained from the Gülhane Health Sciences University Scientific Research Ethics Committee on December 5, 2023, with decision number 2023–401. Written informed consent was obtained from all participants before sample collection. Primary gingival fibroblasts were obtained from tissues during surgical treatment of patients with amlodipine‐associated GO or from clinically healthy tissues excised during crown‐lengthening procedures. Hyperplastic tissue was excised from areas where pseudopockets were ≥ 5 mm in depth after the phase I periodontal treatment in patients receiving amlodipine and experiencing enlargement. Each experimental group consisted of gingival fibroblasts derived from two patients (*n* = 2 per group). Gingival fibroblasts associated with amlodipine‐associated GO were obtained from two male patients aged 52 and 57 years who had been receiving amlodipine therapy for at least 7 years. For the healthy group, gingival fibroblasts were obtained from two systemically healthy male patients aged 49 and 61 years who underwent crown‐lengthening procedures. Growth and healthy gingival tissues containing epithelium and connective tissue were transferred to the laboratory environment using carrier solutions. The epithelial layer was removed from the cell culture medium using a sterile surgical blade, and the tissue fragments were then minced into small pieces of 1 mm^3^ and cultured in 25 cm^2^ flasks containing Dulbecco's Modified Eagle Medium (DMEM) supplemented with 10% fetal bovine serum, 1% penicillin–streptomycin, and 1% L‐glutamine at 37°C in a humidified 5% CO_2_ atmosphere. The medium was refreshed every 72 h until confluence reached approximately 80%. Fibroblasts derived from the two donors within each group were expanded separately to the same passage number and subsequently combined to establish pooled group‐specific cultures. Cells between passages 4 and 7 were used for subsequent experiments and seeded at 1 × 10^5^ cells/well in 12‐well plates.

### 2.2 Cell Viability Assay

Cell viability and proliferation were evaluated using the MTT assay (3‐[4,5‐dimethylthiazol‐2‐yl]‐2,5‐diphenyltetrazolium bromide) at 24 h and 48 h. For MTT assays, cells from individual wells were processed and analyzed separately as technical replicates. MTT measurements were performed in eight assay wells. Following incubation, 20 μL of MTT solution (5 mg/mL in PBS) was added to each well and incubated for 3 h. The supernatant was removed, and 100 μL of isopropanol (0.04 N) was added to dissolve formazan crystals. Absorbance was measured at 570 nm using a microplate reader. Results were normalized to control values and expressed as percentages of viable cells.

### 2.3 Resveratrol Treatment

A stock solution of resveratrol (Sigma, R5010, USA) was prepared by dissolving 11.5 mg of resveratrol in 5 mL DMSO, filtering through a 0.45 μm membrane, and diluting with culture medium to a final concentration of 100 μM. For the 24 h treatment group, resveratrol was applied 24 h post‐seeding; supernatants were collected 24 h later and stored at −80°C for ELISA analysis of inflammatory mediators. For the 48 h group, resveratrol was applied immediately after seeding and again at 24 h; supernatants were collected 48 h after the initial exposure and stored at −80°C.

### 2.4 Biochemical Analyses

Supernatant samples collected at 24 and 48 h and CTGF (Sunlong Biotech CTGF ELISA Kit; sensitivity: 0.01 ng/mL), TGF‐*β*1 (Sunlong Biotech TGF‐*β*1 ELISA Kit; sensitivity: 0.6 pg/mL), COL‐1 (Sunlong Biotech COL‐1 ELISA Kit; sensitivity: 6.5 ng/mL), IL‐6 (Sunlong Biotech IL‐6 ELISA Kit; sensitivity: 0.05 ng/mL), TGM‐2 (Sunlong Biotech TGM‐2 ELISA Kit; sensitivity: 1.2 pg/mL), and SOD (Sunlong Biotech SOD ELISA Kit; sensitivity: 0.05 ng/mL) were evaluated by ELISA according to the manufacturer's instructions. For ELISA assays, supernatants from individual wells were collected separately and analyzed as technical replicates. ELISA samples were measured across five assay wells.

### 2.5 Statistical Analyses

All statistical analyses were performed using a commercially available software program (IBM SPSS Statistics for Windows, version 26.0; IBM Corp., Armonk, NY, USA). The Shapiro–Wilk test was used to assess whether the data followed a normal distribution. For normally distributed variables, one‐way analysis of variance (ANOVA) was applied, followed by Tukey's post hoc test for pairwise comparisons. Categorical variables were compared using the chi‐square (χ^2^) test. Correlations among biochemical parameters were evaluated using Pearson's correlation analysis. A repeated measures General Linear Model (GLM) with a within‐subjects factor (time: 24 h vs. 48 h) and a between‐subjects factor (treatment group) was employed to evaluate temporal changes and group differences across **seven** cellular parameters: cell viability (MTT assay), TGF‐*β*1 expression, CTGF expression, IL‐6 levels, collagen type‐I expression, SOD activity, and TGM‐2 expression. Mauchly's test of sphericity was applied to assess the sphericity assumption for within‐subjects effects. When sphericity was violated (*p* < 0.05), Greenhouse–Geisser corrections were used to adjust the degrees of freedom. Box's M test was performed to evaluate the equality of covariance matrices across groups. For statistically significant main effects or interactions (*α* = 0.05), post hoc pairwise comparisons were conducted using Bonferroni correction to control for Type I error. Effect sizes were reported as partial eta squared (*η2p*), and statistical power was calculated to ensure adequate sensitivity for detecting true effects. A *p* < 05 was considered statistically significant for all analyses.

## 3 Results

### 3.1 MTT Assay Results

**FIGURE 1:**
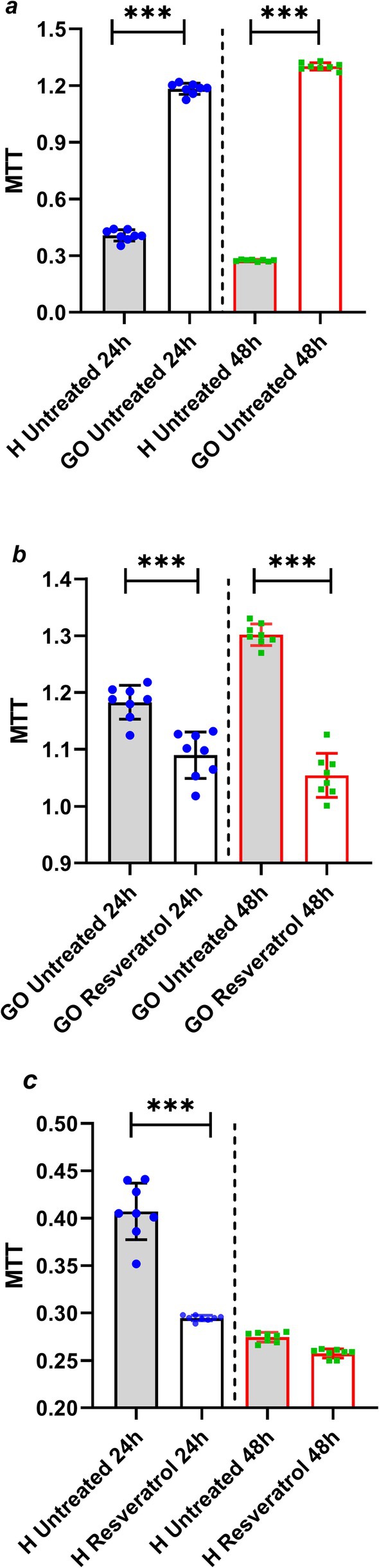
a. Comparison of MTT levels between healty untreated group and gingival overgrowth untreated group. b. Comparison of MTT levels between gingival overgrowth untreated group and gingival overgrowth resveratrol group. c. Comparison of MTT levels between healthy untreated and healthy resveratrol group. Data are presented as mean ± SD.p. *Note*: Statistical significance is indicated as follows: ***p < 0.001.

The MTT findings for gingival fibroblasts in the GO and H groups are presented in (Table 1 and Figure 1). At both 24 and 48 h, MTT values in the GO untreated group were significantly higher than those in the H untreated group (*p* < 0.001). Within the GO untreated group, MTT values at 48 h were significantly higher than at 24 h (*p* = 0.01). In the GO resveratrol‐treated group, although MTT values decreased at 48 h compared with 24 h, the difference was not statistically significant (*p* > 0.05). In the H untreated and resveratrol‐treated groups, MTT values decreased significantly at 48 h compared with 24 h (*p* = 0.01). When the GO untreated and GO resveratrol‐treated groups were compared, MTT values in the GO untreated group were significantly higher at both 24 and 48 h (*p* < 0.001). At 24 h, proliferation values in the H untreated group were significantly higher than those in the H resveratrol‐treated group (*p* < 0.001). At 48 h, no statistically significant difference in MTT values was observed between the H untreated and H resveratrol‐treated groups (*p* > 0.05).

**TABLE 1:** Comparison of MTT assay results between groups.

MTT	H untreated (a) mean ± SD	GO untreated (b) mean ± SD	H resveratrol (c) mean ± SD	GO resveratrol (d) mean ± SD	*p*
24 h	0.407 ± 0.030	1.183 ± 0.030	0.295 ± 0.003	1.090 ± 0.041	a‐b: *p* < 0.0001, a‐c: *p* < 0.0001, b‐d: *p* < 0.0001
48 h	0.274 ± 0.005	1.301 ± 0.018	0.257 ± 0.004	1.054 ± 0.038	a‐b: *p* < 0.0001, a‐c: *p* = 0.499, b‐d: *p* < 0.0001
*p*	*p* = 0.01	*p* = 0.01	*p* = 0.01	*p* > 0.05	

Abbreviations: GO: Gingival overgrowth group; H: Healthy group; SD: Standard deviation.

### 3.2 ELISA Assay Results

**FIGURE 2:**
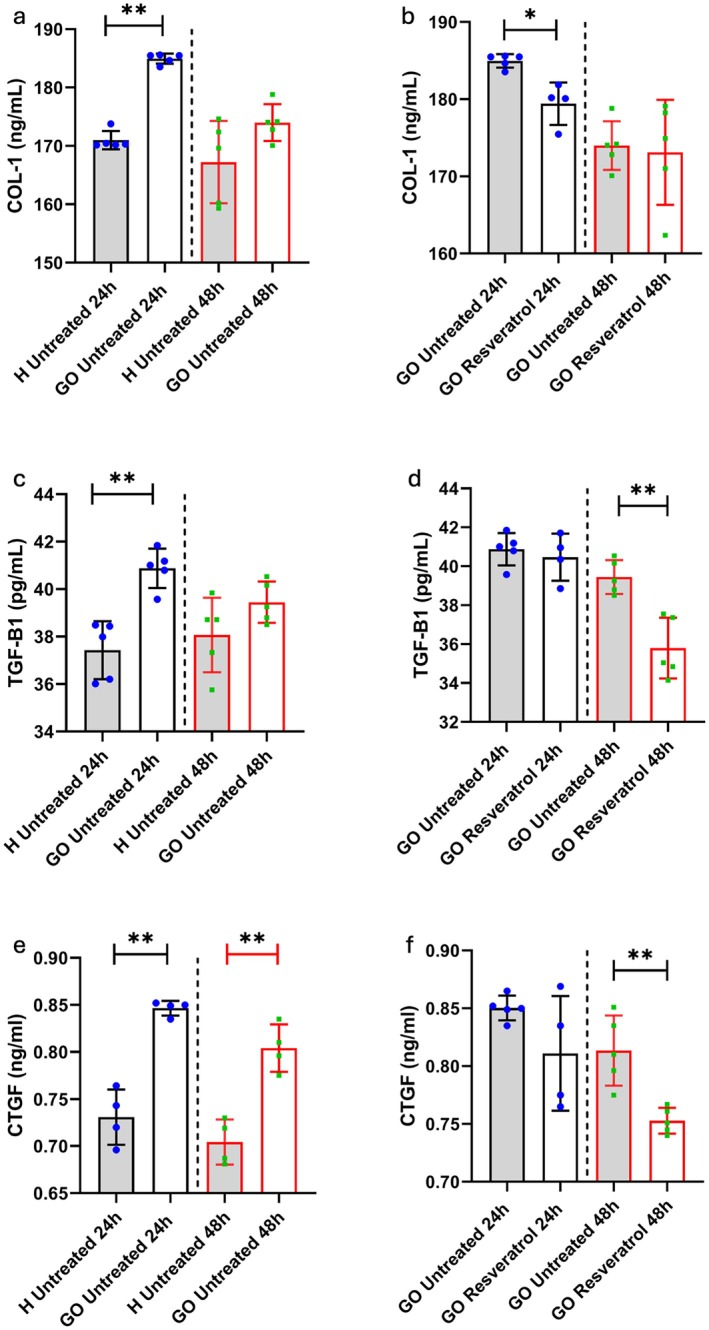
a. Comparison of COL‐1 levels between healty untreated and gingival overgrowth untreated group. b. Comparison of COL‐1 levels between gingival overgrowth untreated group and gingival overgrowth resveratrol group. c. Comparison of TGF‐ β levels between healty untreated and gingival overgrowth untreated group. d. Comparison of TGF‐ *β* levels between gingival overgrowth untreated group and gingival overgrowth resveratrol group (GOR). e. Comparison of CTGF levels between healty untreated and gingival overgrowth untreated group. f. Comparison of CTGF levels between gingival overgrowth untreated group and gingival overgrowth resveratrol group Data are presented as mean ± SD.*Note*: Statistical significance is indicated as follows: *p < 0.05, **p < 0.01.

**FIGURE 3:**
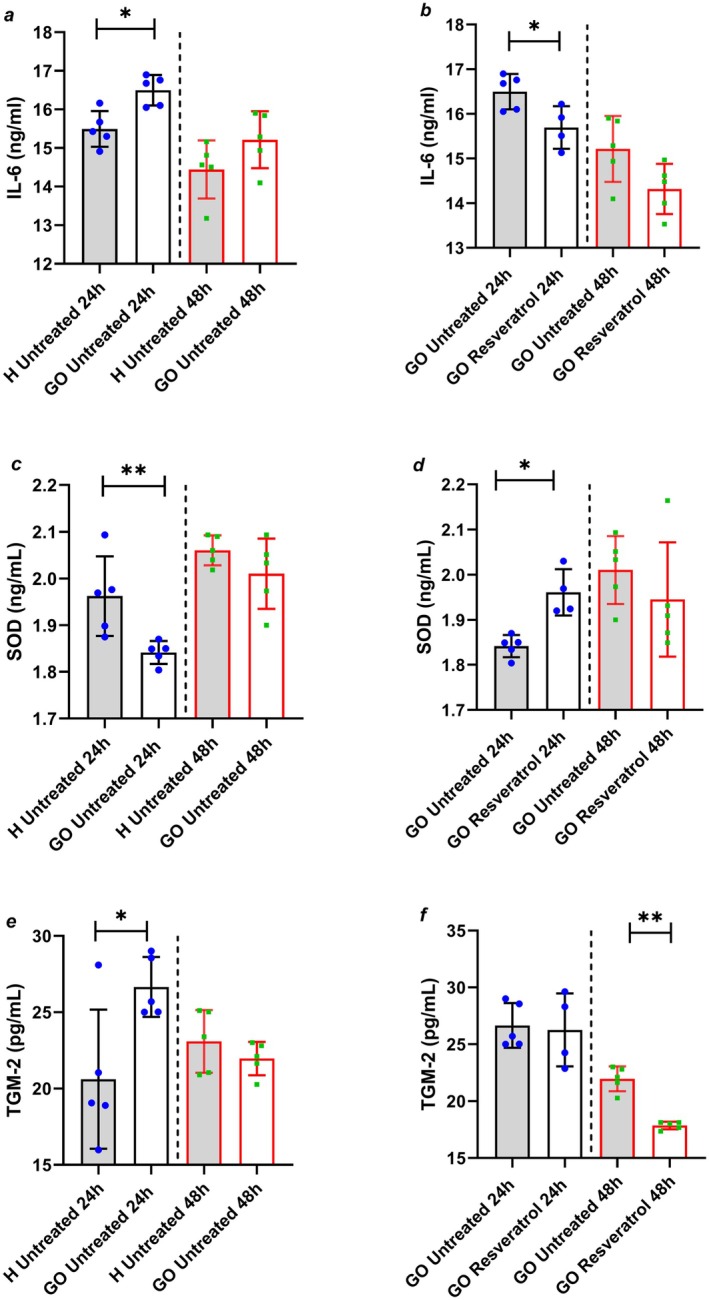
a. Comparison of IL‐6 levels between healty untreated and gingival overgrowth untreated group. b. Comparison of IL‐6 levels between gingival overgrowth untreated group and gingival overgrowth resveratrol group. c. Comparison of SOD levels between healty untreated and gingival overgrowth untreated group. d. Comparison of SOD levels between gingival overgrowth untreated group and gingival overgrowth resveratrol group. e. Comparison of TGM‐2 levels between healty untreated and gingival overgrowth untreated group. f. Comparison of TGM‐2 levels between gingival overgrowth untreated group and gingival overgrowth resveratrol group. Data are presented as mean ± SD.*Note*: Statistical significance is indicated as follows: *p < 0.05, **p < 0.01.

**FIGURE 4:**
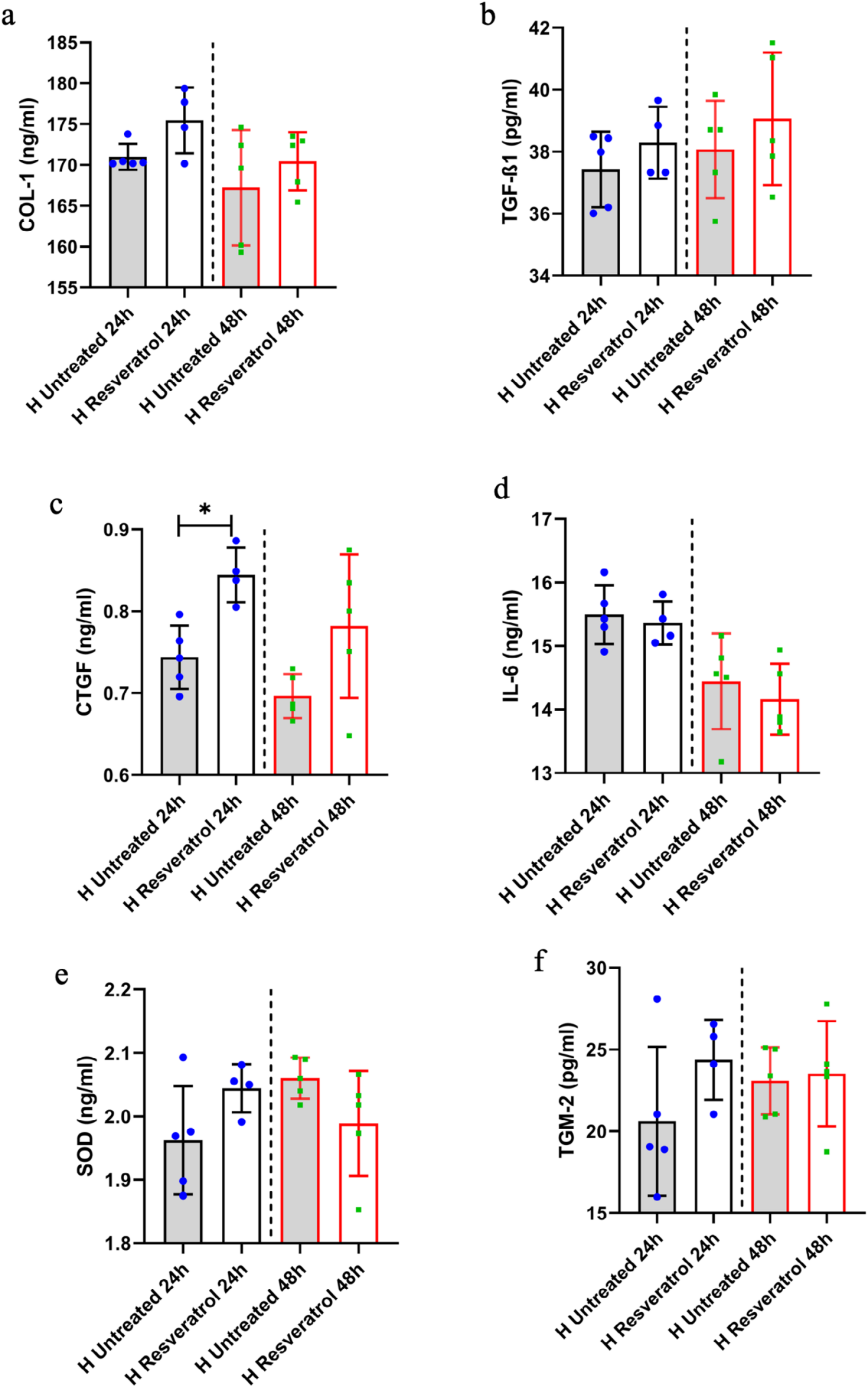
a. Comparision of COL‐1 levels between healthy untreated and healthy resveratrol group b. Comparision of TGF‐ß1 levels between healthy untreated and healthy resveratrol group c. Comparision of CTGF levels between healthy untreated and healthy resveratrol group d. Comparision of IL‐6 levels between healthy untreated and healthy resveratrol group e. Comparision of SOD levels between healthy untreated and healthy resveratrol group f. Comparision of TGM‐2 levels between healthy untreated and healthy resveratrol group. Data are presented as mean ± SD.*Note*: Statistical significance is indicated as follows: *p < 0.05.

The ELISA findings for the H and GO untreated groups are presented in Table 2, and those for the H and GO resveratrol‐treated groups are shown in Table 3. Group comparisons of TGF‐*β*1, CTGF, and COL‐1 ELISA levels are illustrated in Figure 2. At 24 h, COL‐1 levels were significantly higher in GO untreated fibroblasts than in H untreated fibroblasts (*p* < 0.01). Additionally, COL‐1 levels at 24 h were significantly reduced in the GO resveratrol‐treated group compared with the GO untreated group (*p* < 0.05). At 24 h, TGF‐*β*1 levels in the GO untreated fibroblasts were significantly higher than those in the H untreated fibroblasts (*p* < 0.05). At 48 h, TGF‐*β*1 levels in the GO resveratrol‐treated group were significantly reduced compared with the GO untreated group (*p* < 0.01). CTGF levels were significantly higher in the GO untreated group than in the H untreated group at both 24 and 48 h (*p* < 0.01 for both). At 48 h, CTGF levels in the GO resveratrol‐treated fibroblasts were significantly lower than those in the GO untreated fibroblasts (*p* < 0.01). Group comparisons of IL‐6, SOD, and TGM‐2 ELISA levels are shown in Figure 3. At 24 h, IL‐6 levels in the GO untreated fibroblasts were significantly higher than in the H untreated cells (*p* < 0.05). IL‐6 levels in the GO resveratrol‐treated group were lower than those in the GO untreated group at 24 h (*p* = 0.05). SOD levels were significantly lower in the GO untreated group than in the H untreated group at 24 h (*p* < 0.01). In contrast, SOD levels in the GO resveratrol‐treated fibroblasts were significantly higher at 24 h compared with the GO untreated fibroblasts (*p* < 0.05). At 24 h, TGM‐2 levels in the GO untreated fibroblasts were significantly higher than in the H untreated fibroblasts (*p* < 0.05). At 48 h, TGM‐2 levels in the GO resveratrol‐treated group were significantly lower than those in the GO untreated group (*p* < 0.01). Comparisons of ELISA data between the healthy untreated and healthy resveratrol‐treated groups are presented in Figure 4. A statistically significant difference was observed only for CTGF levels at the 24‐h time point.

**TABLE 2:** Comparison of ELISA results between untreated groups.

ELISA	H untreated 24 h (a) mean ± SD	H untreated 48 h (b) mean ± SD	GO untreated 24 h (c) mean ± SD	GO untreated 48 h (d) mean ± SD	*p*
COL‐1	170.98 ± 1.57	167.22 ± 7.06	184.95 ± 0.88	173.99 ± 3.15	a‐b: *p* = 0.5, a‐c: *p* = 0.0088, b‐d: *p* = 0.1172, c‐d: *p* = 0.04
TGF‐ *β*1	37.43 ± 1.22	38.07 ± 1.57	40.88 ± 0.83	39.45 ± 0.87	a‐b: *p* = 0.35, a‐c: *p* = 0.0090, b‐d: *p* = 0.1161, c‐d: *p* = 0.08
CTGF	0.74 ± 0.04	0.70 ± 0.03	0.85 ± 0.01	0.81 ± 0.03	a‐b: *p* = 0.08, a‐c: *p* = 0.0090, b‐d: *p* = 0.0090, c‐d: *p* = 0.04
IL‐6	15.50 ± 0.46	14.44 ± 0.75	16.50 ± 0.39	15.21 ± 0.74	a‐b: *p* = 0.04, a‐c: *p* = 0.0283, b‐d: *p* = 0.1172, c‐d: *p* = 0.04
SOD	1.96 ± 0.09	2.06 ± 0.03	1.84 ± 0.02	2.01 ± 0.08	a‐b: *p* = 0.08, a‐c: *p* = 0.0090, b‐d: *p* = 0.2948, c‐d: *p* = 0.04
TGM‐2	20.61 ± 4.55	23.09 ± 2.06	26.66 ± 1.96	21.97 ± 1.09	a‐b: *p* = 0.27, a‐c: *p* = 0.0472, b‐d: *p* = 0.3472, c‐d: *p* = 0.04

Abbreviations: COL‐1, Collagen type 1; CTGF, Connective tissue growth factor; GO, Gingival overgrowth group; H, Healthy group; IL‐6, Interleukin‐6; SD, Standard deviation; SOD, Superoxide dismutase; TGF‐*β*1, Transforming growth factor‐ *β*1; TGM‐2, Transglutaminase 2.

**TABLE 3:** Comparison of ELISA results between resveratrol groups.

ELISA	H resveratrol 24 h (a) mean ± SD	H resveratrol 48 h (b) mean ± SD	GO resveratrol 24 h (c) mean ± SD	GO resveratrol 48 h (d) mean ± SD	*p*
COL‐1	175.46 ± 4.04	170.44 ± 3.57	179.40 ± 2.75	173.12 ± 6.80	a‐b: *p* = 0.14, a‐c: *p* = 0.0833, b‐d: *p* = 0.3472, c‐d: *p* = 0.07
TGF‐ *β*1	38.29 ± 1.16	39.06 ± 2.14	40.46 ± 1.21	35.79 ± 1.56	a‐b: *p* = 0.47, a‐c: *p* = 0.0575, b‐d: *p* = 0.0283, c‐d: *p* = 0.07
CTGF	0.84 ± 0.03	0.78 ± 0.09	0.81 ± 0.05	0.75 ± 0.01	a‐b: *p* = 0.14, a‐c: *p* = 0.2482, b‐d: *p* = 0.2948, c‐d: *p* = 0.07
IL‐6	15.36 ± 0.34	14.17 ± 0.56	15.69 ± 0.47	14.32 ± 0.56	a‐b: *p* = 0.07, a‐c: *p* = 0.2482, b‐d: *p* = 0.6015, c‐d: *p* = 0.07
SOD	2.04 ± 0.04	1.99 ± 0.08	1.96 ± 0.05	1.94 ± 0.13	a‐b: *p* = 0.27, a‐c: *p* = 0.0433, b‐d: *p* = 0.3472, c‐d: *p* = 1.00
TGM‐2	24.37 ± 2.45	23.52 ± 3.22	26.26 ± 3.20	17.85 ± 0.33	a‐b: *p* = 0.27, a‐c: *p* = 0.3865, b‐d: *p* = 0.0088, c‐d: *p* = 0.07

Abbreviations: COL‐1, Collagen type 1; CTGF, Connective tissue growth factor; GO, Gingival overgrowth group; H, Healthy group; IL‐6, Interleukin‐6; SD, Standard deviation; SOD, Superoxide dismutase; TGF‐*β*1, Transforming growth factor‐*β*1; TGM‐2, Transglutaminase 2.

A repeated measures GLM analysis was conducted to evaluate seven cellular parameters across four experimental groups (GO Resveratrol, GO Untreated, H Resveratrol, and H Untreated) at two time points (24 h and 48 h). The MTT assay revealed a highly significant group × time interaction (F = 46.507, *p* < 0.001, *η2p* = 0.833), indicating differential temporal changes in cell viability among treatment groups, with a large effect size. In addition, between‐group differences were highly significant (F = 9726.925, *p* < 0.001, *η2p* = 0.999), confirming a pronounced treatment‐related effect on cellular viability.

TGF‐*β*1 expression demonstrated a significant group × time interaction (F = 5.600, *p* = 0.010, *η2p* = 0.545) accompanied by significant between‐group differences (F = 6.679, *p* = 0.005, *η2p* = 0.589), with GO Untreated samples exhibiting significantly higher levels compared to H Untreated (*p* = 0.005). The main effect of time did not reach statistical significance but showed a trend toward significance (F = 4.086, *p* = 0.063, *η2p* = 0.226).

CTGF analysis revealed significant main effects of time (F = 12.751, *p* = 0.003, *η2p* = 0.477) and group (F = 11.653, *p* < 0.001, *η2p* = 0.714), both with large effect sizes, whereas no significant interaction was observed (F = 0.202, *p* = 0.893, *η2p* = 0.041), indicating parallel temporal patterns across groups. Post hoc Bonferroni comparisons showed that GO Untreated samples had significantly higher CTGF expression than H Untreated samples (*p* < 0.001).

IL‐6 levels exhibited a strong main effect of time (F = 43.206, *p* < 0.001, *η2p* = 0.755) and significant between‐group differences (F = 9.343, *p* = 0.001, *η2p* = 0.667), both representing large effect sizes. However, no significant group × time interaction was detected (F = 0.152, *p* = 0.927, *η2p* = 0.031), suggesting consistent temporal increases across all experimental conditions.

Collagen Type‐I expression showed significant effects of time (F = 19.589, *p* = 0.001, *η2p* = 0.583) and group (F = 15.156, *p* < 0.001, *η2p* = 0.765), with GO Untreated samples displaying significantly higher expression compared to GO Resveratrol (*p* = 0.044).

SOD activity demonstrated a significant group × time interaction (F = 4.436, *p* = 0.022, *η2p* = 0.487), despite non‐significant main effects of time (F = 3.184, *p* = 0.096, *η2p* = 0.185) and group (F = 3.193, *p* = 0.057, *η2p* = 0.406), indicating group‐specific temporal modulation of antioxidant activity.

Finally, TGM‐2 expression exhibited a significant main effect of time (F = 14.383, *p* = 0.002, *η2p* = 0.507) along with a pronounced group × time interaction (F = 9.488, *p* = 0.001, *η2p* = 0.670), whereas overall between‐group differences were not statistically significant (F = 1.435, *p* = 0.275, *η2p* = 0.235).

## 4 Discussion

In this study, we aimed to determine whether resveratrol exhibited an anti‐proliferative effect on gingival fibroblasts derived from GO, associated with amlodipine use in hypertensive patients. We sought to investigate how resveratrol influenced the levels of key mediators involved in collagen and ECM metabolism and associated with increased fibrotic susceptibility, including the growth factors TGF‐*β* and CTGF, as well as type I collagen, IL‐6, TGM‐2, and SOD.

There has been growing interest in studies examining polyphenols that demonstrate anti‐fibrotic properties in various organs and tissues under fibrotic conditions. Resveratrol, one such polyphenol, is known for its anti‐inflammatory, antimicrobial, cardioprotective, vasoprotective, and antioxidant effects [21]. Several studies have reported the potential use of resveratrol in the development of non‐invasive therapeutic approaches for the treatment of fibrotic tissues in different organs [24, 26, 27, 28]. The first report demonstrating the anti‐proliferative effect of resveratrol on genetically driven gingival fibromatosis was a study using gingival fibroblasts cultured from hyperplastic tissues of a patient with juvenile hyaline fibromatosis [13]. In studies investigating how amlodipine influences the fibrotic response, gingival fibroblasts incubated with amlodipine have typically been derived either from commercial cell lines or from healthy tissues exposed to the drug under in vitro conditions [29]. The present study is the first to use primary fibroblast cultures obtained directly from hyperplastic tissues of patients with amlodipine‐induced GO. The 100 μM concentration of resveratrol selected to evaluate its anti‐proliferative effect on gingival fibroblasts cultured from hyperplastic tissues was based on our previous study, in which we demonstrated that resveratrol exerted an anti‐proliferative effect on fibroblasts derived from primary cultures of excessively enlarged gingival tissues in a patient with juvenile hyaline fibromatosis [13]. The most substantial effects were observed at 100 μM and 200 μM concentrations. 100 μM was chosen for use in the present study to minimize potential cytotoxicity while maintaining efficacy. Cell proliferation and cytotoxicity in all experimental groups were evaluated at 24 and 48 h using repeated MTT assays. When the MTT values of gingival fibroblasts obtained from enlarged gingival tissues of patients who had been using amlodipine for at least 7 years were compared with those of healthy control fibroblasts, proliferation levels were found to be significantly higher at both 24 and 48 h. Overgrowth fibroblasts exhibited markedly increased proliferation at both time points, with the difference becoming even more pronounced at 48 h. In gingival fibroblasts cultured from enlarged gingival tissues of a patient with juvenile hyaline fibromatosis, a genetically determined condition, MTT assays performed at 24, 48, and 72 h demonstrated a marked time‐dependent increase in cellular proliferation. This proliferative pattern was considered a characteristic feature similar to that observed in fibroblasts derived from amlodipine‐induced gingival overgrowth. In contrast, cells obtained from healthy gingival tissues exhibited a progressive decline in proliferation over time. These findings suggested that fibroblasts derived from amlodipine‐associated hyperplastic tissues possessed distinct phenotypic characteristics compared with healthy gingival fibroblasts. McKevitt et al. demonstrated phenotypic differences between gingival fibroblasts isolated from two patients receiving long‐term nifedipine therapy who exhibited gingival overgrowth and fibroblasts obtained from clinically healthy gingiva of similarly treated individuals. They reported that cells derived from enlarged tissues had greater proliferative capacity and produced higher levels of protein and collagen than fibroblasts isolated from non‐enlarged tissues [30]. In this study, resveratrol did not exhibit anti‐proliferative or cytotoxic effects on healthy gingival fibroblasts. However, in the overgrowth group, resveratrol treatment significantly reduced cell proliferation at both 24 and 48 h compared with GO untreated fibroblasts, without inducing cytotoxicity. This study is the first to report that resveratrol suppresses the excessive proliferation of fibroblasts derived from drug‐induced gingival overgrowth without exerting cytotoxic effects. Similarly, resveratrol has previously been shown to exert anti‐proliferative effects on gingival fibroblasts obtained from gingival overgrowth associated with genetic conditions such as juvenile hyaline fibromatosis [13]. Although similar experimental endpoints were employed, the two disease models differ fundamentally in etiology and inflammatory burden. Therefore, comparable response patterns to resveratrol across these models should be interpreted as disease‐context validation rather than direct equivalence between fibrotic entities.

The exact mechanism underlying amlodipine‐associated gingival enlargement remains unclear; however, it is thought to result from interactions among gingival fibroblasts, cellular and biochemical mediators of inflammation, and drug metabolites [3, 31]. Because increased ECM accumulation has been reported in drug‐induced gingival overgrowth [32] research has focused on the interaction between the drug and the resident fibroblast populations as a key underlying mechanism. Phenotypic heterogeneity within resident fibroblast populations of a given tissue has also been documented in the literature [33]. One of the most important cytokines regulating collagen metabolism is TGF‐*β*, which plays a key role in modulating fibroblast proliferation, collagen and ECM synthesis, activation of TIMPs, inhibition of MMPs, and other characteristic processes observed in fibrotic lesions [34]. Comparison of TGF‐*β*1 levels across groups revealed that, at 24 h, TGF‐*β*1 levels were significantly higher in overgrowth fibroblasts than in healthy control fibroblasts. At 48 h, TGF‐*β*1 levels in the overgrowth group remained higher, although the difference was not statistically significant. Repeated resveratrol administration reduced TGF‐*β*1 levels in the overgrowth fibroblasts compared with untreated overgrowth fibroblasts, while resveratrol did not affect TGF‐*β*1 expression in healthy gingival fibroblasts. Consistent with our findings, resveratrol has also been reported to reduce TGF‐*β*1 levels in gingival fibroblasts derived from juvenile hyaline fibromatosis–associated gingival enlargement [13]. Numerous studies have examined the anti‐fibrotic effects of resveratrol in other organ fibrosis models as well. For example, in rheumatoid arthritis–associated pulmonary fibrosis, resveratrol has been shown to decrease TGF‐*β*1 levels [35]. Similarly, reductions in TGF‐*β*1 following resveratrol treatment have been reported in rat models of pulmonary, myocardial, and renal fibrosis [36, 37]. Jin Lv et al. also demonstrated that resveratrol reduced TGF‐*β*1 levels and improved urethral fibrosis in a rat model of TGF‐*β*1–mediated urethral fibrosis [38]. Given that TGF‐*β* is considered a key molecular regulator of fibrogenesis [10, 34], the observed suppression of TGF‐*β*1 levels in gingival fibroblasts derived from amlodipine‐associated gingival enlargement suggests that resveratrol may exert an anti‐fibrotic effect in hyperplastic gingival tissues.

CTGF, a multifunctional matricellular protein, has been reported to be elevated in various fibrotic pathologies [39] and is also highly expressed in fibrotic lesions of gingival tissues [9]. In a study investigating CTGF levels in amlodipine‐induced gingival hyperplasia, hypertensive patients using amlodipine were divided into two groups based on the presence or absence of gingival overgrowth; CTGF gene expression in gingival tissues was found to be higher in the group exhibiting gingival enlargement [3]. CTGF levels have been shown to correlate positively with fibrosis and to play a role in stimulating and maintaining fibrotic processes [40]. CTGF levels were significantly higher in gingival fibroblasts from the overgrowth group compared with healthy control fibroblasts. This finding is consistent with previous studies reporting elevated CTGF levels in amlodipine‐associated gingival enlargement [3]. Although no significant difference in CTGF levels was observed between the H resveratrol‐treated and GO resveratrol‐treated groups, this result is important because it indicates that the markedly elevated CTGF levels in overgrowth fibroblasts were reduced to levels comparable to those of healthy fibroblasts following resveratrol treatment. CTGF levels in resveratrol‐treated overgrowth fibroblasts were lower than those in untreated overgrowth cells at 24 h, and the reduction became statistically significant at 48 h when comparing the GO resveratrol‐treated and GO untreated groups. These findings demonstrate that repeated resveratrol administration decreases CTGF levels—one of the key mediators of gingival overgrowth [9, 12, 13]. Consistent with our results, decreased CTGF expression following resveratrol treatment has also been reported in gingival fibroblasts derived from a patient with juvenile hyaline fibromatosis [13]. Studies in other organ fibrosis models similarly support this effect; for example, CTGF levels were reduced following resveratrol administration in rat models of renal fibrosis [37], and pulmonary fibrosis [25]. The ability of resveratrol to lower CTGF levels in gingival fibroblasts derived from amlodipine‐associated overgrowth, without adversely affecting CTGF synthesis in healthy fibroblasts, suggests that resveratrol may exert beneficial effects without disrupting tissue homeostasis.

Regarding COL‐1 levels, we found that gingival fibroblasts from the overgrowth group expressed significantly higher COL‐1 compared with healthy fibroblasts. Resveratrol treatment reduced COL‐1 levels in fibroblasts derived from gingival overgrowth, whereas no change was observed in COL‐1 synthesis in healthy cells. In the study conducted by Kaleci and Koyutürk, which investigated wound healing in a Swiss albino rat model, resveratrol did not induce a statistically significant alteration in COL1A2 mRNA expression levels. This observation is in agreement with the COL‐1 expression findings obtained from healthy gingival fibroblasts in the present study [41]. Consistent with our findings, resveratrol has also been reported to decrease COL‐1 levels in gingival fibroblasts obtained from a patient with juvenile hyaline fibromatosis as well as in other fibrosis models [13]. Lei Liu et al. demonstrated reduced COL‐1 expression in a rat model of pulmonary fibrosis [25], and Jin Lv et al. observed similar reductions in COL‐1 levels in a rat model of urethral fibrosis [38].

IL‐6 plays roles in inflammation, immune responses, hematopoiesis, and ECM production, and has been reported to contribute to the inflammatory component of calcium channel blocker–associated gingival enlargement [42]. IL‐6 levels were higher in overgrowth fibroblasts than in healthy fibroblasts. Resveratrol treatment reduced IL‐6 levels in overgrowth cells, with this reduction approaching statistical significance. Because no previous study has assessed the effect of resveratrol on IL‐6 synthesis in gingival enlargement, we interpreted our findings in light of research examining the relationship between this cytokine and resveratrol in other disease models. For example, resveratrol was reported to decrease IL‐6 levels in endometrial stromal cells obtained from patients with endometriosis [43]. Chowdhury et al. also demonstrated that resveratrol reduced elevated IL‐6 levels in a Wistar rat model of diet‐induced renal fibrosis [44]. Although IL‐6 levels decreased following resveratrol treatment in our study, the difference did not reach statistical significance. We speculate that initial periodontal therapy performed prior to crown‐lengthening or gingivectomy procedures in patients with amlodipine‐associated gingival enlargement may have reduced baseline IL‐6 levels—given that IL‐6 is a pro‐inflammatory cytokine—thereby limiting the magnitude of detectable differences among the groups.

SOD is a cellular antioxidant enzyme, and reduced SOD levels have been reported in various organ fibrosis models [19]. In our study, SOD levels were lower in the overgrowth group compared with healthy fibroblasts. This reduction was statistically significant at 24 h; however, SOD levels remained lower at 48 h, and the difference was not statistically significant. Mojsilovic et al. reported higher SOD activity in gingival tissues exhibiting amlodipine‐associated gingival enlargement [3]. This discrepancy may be attributed to methodological differences, as our study assessed SOD concentration using an ELISA method, whereas the previous study measured enzymatic activity using a colorimetric assay. Consistent with the decreased SOD levels observed in overgrowth fibroblasts in our study, several fibrosis models have reported reductions in SOD and catalase content accompanied by increased malondialdehyde levels [45, 46]. In the overgrowth group, resveratrol treatment resulted in significantly higher SOD levels at 24 h compared with GO untreated, although no significant difference was observed at 48 h. No prior study has investigated the relationship between resveratrol and SOD levels in gingival enlargement. Chowdhury et al. demonstrated that resveratrol restored SOD activity and gene expression in Wistar rats with diet‐induced renal fibrosis, in which a high‐fat diet reduced SOD levels [44].

TGM‐2 is an enzyme reported to contribute to fibrosis through ECM accumulation in the kidney and liver and to be activated by ROS [14]. Elevated TGM‐2 levels have also been reported in cyclosporine‐associated gingival overgrowth [47, 48]; however, no studies to date have investigated its role in calcium channel blocker–induced gingival enlargement. In our study, TGM‐2 levels were evaluated for the first time in this context. At 24 h, TGM‐2 levels were significantly higher in overgrowth fibroblasts than in healthy cells, whereas no statistically significant difference was observed at 48 h. Repeated resveratrol administration significantly reduced TGM‐2 levels in overgrowth fibroblasts but did not affect healthy gingival fibroblasts. Only a limited number of studies have examined the relationship between resveratrol and TGM‐2, and existing findings are inconsistent. For instance, an in vitro study on human cholangiocarcinoma cells reported that resveratrol increased TGM‐2 activity [49] whereas another study in human hepatocarcinoma cells found that resveratrol did not alter TGM‐2 expression [50]. Therefore, further in vitro and experimental studies are needed to clarify the effects of resveratrol on TGM‐2 regulation in gingival fibrosis.

One major strength of this study is the use of primary fibroblasts cultured directly from hyperplastic gingival tissues of patients receiving amlodipine. These overgrowth fibroblasts exhibited distinct phenotypic features compared with healthy controls, supporting their relevance as a more accurate model for evaluating drug–cell interactions. Another strength is the repeated evaluations at 24 and 48 h, which enabled assessment of temporal changes in mediators related to proliferation and hyperplasia. Repeated MTT and ELISA analyses further demonstrated that consecutive resveratrol dosing possibly exerted more substantial anti‐fibrotic and anti‐proliferative effects on overgrowth fibroblasts. This study also has limitations. A primary limitation of the present study is the absence of direct cell number normalization for mediator levels measured in culture supernatants. Consequently, changes in cytokine and fibrosis‐related markers cannot be interpreted as strictly per‐cell effects. Although resveratrol reduced proliferation in overgrowth fibroblasts at the cellular level, long‐term clinical studies are required before considering it as a therapeutic option for gingival hyperplasia. Additionally, both overgrowth and healthy control tissues were obtained after initial periodontal therapy, which may have reduced inflammatory status and influenced mediator levels.

## 5 Conclusions

The overgrowth fibroblasts exhibited markedly higher proliferation than healthy fibroblasts, suggesting distinct phenotypic characteristics. In amlodipine‐associated gingival enlargement, treatment duration, dosage, the presence of inflammation, and individual genetic susceptibility are considered important risk factors for the development of gingival hyperplasia; therefore, fibroblasts derived directly from hyperplastic tissues exposed to these factors may represent a more appropriate model for evaluating drug–cell interactions. In fibroblasts obtained from hyperplastic gingival tissues, resveratrol exerted beneficial effects by suppressing excessive proliferation without inducing cytotoxicity, reducing the abnormal levels of TGF‐*β*1, CTGF, COL‐1, and TGM‐2, lowering IL‐6 levels, and increasing SOD levels.

## Funding

This research did not receive any specific grant from funding agencies in the public, commercial, or not‐for‐profit sectors.

## Disclosure

AI Statement: This manuscript used NLP (ChatGPT) for proofreading an entirely human‐generated text, but no other use of AI was made. Permission to Reproduce Material From Other Sources: No previously published figures, tables, or materials were reproduced in this manuscript; therefore, no permissions were required.

## Ethics Statement

Ethical approval for this study was obtained from the Gülhane Health Sciences University Scientific Research Ethics Committee.

## Consent

Written informed consent was obtained from all tissue donors prior to sample collection.

## Conflicts of Interest

The authors declare no conflicts of interest.

## Data Availability

The data that support the findings of this study are available on request from the corresponding author. The data are not publicly available due to privacy or ethical restrictions.
